# Diagnostic Biomarkers for Invasive Candidiasis: A Clinician-Oriented Review

**DOI:** 10.3390/jof12010055

**Published:** 2026-01-12

**Authors:** Sebastian George Smadu, Simona Camelia Tetradov, Luminita Ene, Corina Oprisan, Dragoș Ștefan Lazăr, Simin Aysel Florescu

**Affiliations:** 1 Department of Infectious Diseases, “Carol Davila” University of Medicine and Pharmacy, 37 Dionisie Lupu Street, 020021 Bucharest, Romania; sebastian-george.smadu@drd.umfcd.ro (S.G.S.); corina.barbu@drd.umfcd.ro (C.O.); dragos.lazar@umfcd.ro (D.Ș.L.); simin.florescu@umfcd.ro (S.A.F.); 2 “Victor Babes” Clinical Hospital for Infectious and Tropical Diseases, 281 Mihai Bravu Street, 030303 Bucharest, Romania; luminita.ene@spitalulbabes.ro

**Keywords:** invasive candidiasis, 1-3-β-D-Glucan, PCR *Candida* species, mannan, anti-mannan, invasive candidiasis diagnostic pathway

## Abstract

Introduction: A group of approximately 15 Candida species are frequently found to be responsible for human invasive candidiasis, an infection that appears in patients with prolonged hospitalization, particularly in Intensive Care Units, and in immunosuppressed individuals. Given the considerable burden if not rapidly treated, clinicians face diagnostic challenges in distinguishing infection. The objective of this narrative review is to summarize the clinically applicable biomarkers used for invasive candidiasis and to evaluate their performance and create a diagnostic algorithm for clinical practice. Methods: This narrative review was conducted by searching PubMed and Scopus for studies published between 1990 and 2025, using keywords related to invasive candidiasis and non-culture diagnostic biomarkers. Clinical guidelines and consensus documents from major infectious diseases societies were additionally reviewed to supplement. Results: Blood cultures, which are considered the “gold standard” for diagnosis, face important fallouts caused by the limited sensitivity of 50%. Polymerase Chain Reaction assays can identify Candida species at an early stage when compared to blood cultures, demonstrating high specificity that ranges between 91% and 98, due to their high cost, and the limitations regarding only the identification of certain species, their widespread use remains limited. Non-culture serological tests such as mannan, anti-mannan and 1-3-β-D-glucan can detect fungal cell wall components or antibodies directed towards them. These tests have the advantage of being performed directly from blood samples. Reported sensitivity and specificity are 83% and 86% for mannan/anti-mannan, and 73% and 80% for 1-3-β-D-glucan, respectively. They are used for early detection of candidemia in high-risk patients, including immunocompromised individuals. Conclusions: Our report suggests that the traditional “gold standard” for diagnosing invasive candidiasis can be improved by integrating and combining novel biomarkers in the diagnostic pathways, and, thus, potentially reducing the time spent for diagnosing and facilitating early treatment access.

## 1. Introduction

Of the 150 identified *Candida* species, approximately 15 are potentially harmful in humans. Most invasive infections are attributed to a few species: *Candida albicans*, *Nakaseomyces glabratus* (formerly *Candida glabrata*), *Candida parapsilosis*, *Candida tropicalis*, and *Pichia kudriavzevii* (formerly *Candida krusei*). In recent years, *Candidozyma auris* (formerly *Candida auris*) has emerged as an important pathogen, particularly due to its ability to spread within healthcare settings [1,2].

Well-known risk factors for developing invasive candidiasis are surgery, indwelling catheters, diabetes, total parenteral nutrition, and prolonged hospital stay, especially in the Intensive Care Unit (ICU). With the widespread adoption of novel immunosuppressive therapies in cancer patients, myelodysplastic syndromes, and rheumatological diseases, the burden of fungal infections has increased. As the population is aging, patients accumulate multiple risk factors, exposing them to new threats, including *Candida* infections [3]. Recognizing these risk factors helps identify when biomarker testing is most useful, particularly in high-risk patients. The main predisposing conditions are presented in Table 1.

Despite antifungal therapy and the development of new antifungal drugs, invasive candidiasis continues to carry a high mortality burden, often ranging from 40% to 60% or even higher in some immunocompromised cohorts [8].

High mortality of *Candida* infections is due to its inherent invasiveness. Patients with immune defects have a high mortality, and those lacking traditional risk factors also experience diagnostic delays [9].

The pathogenicity of *Candida* is multifactorial, involving several virulence factors, including the ability to switch between yeast and hyphal forms, the capacity to adhere to host tissues, and the excretion of enzymes such as proteases and phospholipases. A major pathogenic mechanism of *Candida* is biofilm formation on biotic and abiotic surfaces, which confers high resistance to both host immune response and antifungal agents. These biofilms are complex volumetric assemblies composed of yeast cells, hyphae, and pseudo-hyphae that are embedded in an extracellular matrix. These fungal networks are responsible for the persistence of infection and treatment failure [10,11,12].

Based on the affected site, *Candida* infections range from superficial, affecting the skin, nails, oral or vaginal mucosa, to invasive or systemic infection involving the bloodstream, eyes, spleen, liver, or central nervous system. Mucocutaneous candidiasis is typically associated with a high morbidity but has a low-to-negligible risk of mortality, in contrast to systemic candidiasis, which is associated with a higher mortality rate [13].

Because of the high burden of invasive candidiasis in patients with cancer or hematological malignancies, in 2019, the European Organization for Research and Treatment of Cancer and the Mycoses Study Group Education and Research Consortium (EORTC/MSGERC) defined invasive fungal infection and updated the previous 2008 definitions, aiming to better incorporate non-culture biomarkers into the diagnostic criteria. The consensus classified invasive candidiasis into three categories: Proven, Probable, and Possible [4].

- Proven Invasive *Candida* Infections are diagnosed based on histopathologic or cytopathologic proof of *Candida* species from a specimen obtained by needle aspiration of biopsy. A culture of the specimen obtained by a sterile procedure from a normally sterile site, such as blood, is confirmatory (e.g., blood cultures) [4].
- Probable Invasive *Candida* Infections are diagnosed with the summation of three factors: host risk factors, suggestive clinical settings, and mycological criteria [4].
  ▪ Host risk factors include the following: recent history of neutropenia defined as high as 500 cells/mm^3^ neutrophils for at least 10 days; hematologic malignancy; recipient of an allogeneic stem cell transplant or solid organ transplant; prolonged use of corticosteroids at the therapeutic dose of equal or more than 0.3 mg/kg of corticosteroids for more than 3 weeks; treatment with other recognized T-cell immunosuppressants during the past ninety days; inherited severe immunodeficiency; and acute graft versus host disease grade III or IV [4].
  ▪ Clinically suggestive of disseminated candidiasis is defined when patients have experienced candidemia within the last two weeks, along with at least one of the following findings: “bull’s eye’ lesions”—small abscesses—seen in the spleen or the liver, or progressive retinal exudates upon ophthalmologic examination [4].
  ▪ Mycological criteria for *Candida* species include a positive 1-3-β-D-Glucan test with a cutoff of 80 pg/mL in at least two consecutive serum samples [4].
- Possible Invasive *Candida* Infections are diagnosed based on the presence of suggestive clinical findings and relevant host factors but in the absence of positive mycological evidence [4].

Although *Candida* species predominantly infect immunocompromised individuals as previously stated, they also substantially contribute to a high morbidity and mortality among patients with prolonged hospital stay, especially those in ICU. In these patients, with no or undiagnosed immunosuppression the diagnostic pathways outlined in EORTC/MSGERC criteria do not apply.

To address this gap, in 2024, Invasive Fungal Diseases in Adult Patients in Intensive Care Unit (FUNDICU) guidelines were created aiming to better diagnose candidemia in ICU patients. According to this consensus, candidemia is confirmed by a peripheral blood culture positive for *Candida* species, representing definitive evidence of infection, regardless of immune status. Positive blood cultures obtained from central venous catheters are excluded from this definition, as catheters can be easily contaminated. The FUNDICU guideline introduces a research-based framework to standardize definitions for invasive fungal infections in ICU patients; but still, its application currently remains primarily for research purposes, rather than for routine clinical use [5].

In 2025 the European Confederation of Medical Mycology in collaboration with the International Society for Human & Animal Mycology and American Society for Microbiology published a joint guideline, highlighting that the diagnosis of candidiasis should rely on a combination of clinical evaluation and confirmatory microbiological methods. Specimens obtained aseptically from normally sterile sites provide definitive evidence of infection when culture or microscopy identifies *Candida* species, regardless of the limited sensitivity of these methods. The same guideline highlights that molecular assays, including commercially available Polymerase Chain Reaction (PCR) tests and serum biomarkers such as 1-3-β-D-Glucan and mannan/anti-mannan antibodies, although offering rapid results, should be interpreted as complementary diagnostic tools along with traditional techniques [7].

Several diagnostic scores that integrate clinical assessment, predisposing factors, and laboratory markers have been proposed to facilitate early identification of invasive candidiasis over the years. However, due to variable sensitivity, specificity, and limited applicability across patient populations, none have achieved widespread validation or have received formal endorsement in international guidelines [7].

Despite the availability of multiple diagnostic assays, early and species-specific detection remains inconsistent in real-world clinical practice.

The aim of this narrative review is to provide a clinically applicable overview of diagnostic tools for invasive candidiasis, to facilitate clinical decision making, and to outline a clinician-orientated diagnostic algorithm integrating both culture-based and non-culture-based biomarkers.

## 2. Materials and Methods

We conducted a literature search in PubMed and Scopus for the following keywords as search strings or for creating specific queries: “invasive candidiasis”, “candidemia”, “deep-seated candidemia”, “candida biomarker”, “1-3-β-D-Glucan”, “Mannan Antigen”, “Anti-mannan antibodies”, “PCR *Candida*”, and “serology”.

We assessed studies published between 1990 and 2025. Articles from 1990 to 2000 were particularly focused on classical microbiological diagnostic methods, especially blood culture techniques.

We included in this review only English language studies which evaluated non-culture biomarkers used for diagnosing invasive candidiasis, that reported diagnostic performance information (Sensitivity, Specificity, Positive Predictive Values, Negative Predictive Values) and that used culture-based and/or histopathological methods as reference.

We excluded single patient case reports, animal studies, and papers without clinically applicable information.

As this is a narrative review, the search strategy was designed to identify clinically relevant diagnostic evidence rather than to comprehensively retrieve all published studies.

Information provided from the search was supplemented by integrating guidelines and consensus statements from the European Society of Clinical Microbiology and Infectious Diseases Guidelines (ESCMID), Infectious Diseases Society of America Guidelines (IDSA), European Organization for Research and Treatment of Cancer/Invasive Fungal Infections Cooperative Group and the National Institute of Allergy and Infectious Diseases Mycoses Study Group (EORTC/MSG), Fungal Infections Definitions in Intensive Care Unit Patients (FUNDICU), European Confederation of Medical Mycology (ECMM), International Society for Human & Animal Mycology (ISHAM), and the American Society for Microbiology (ASM).

This paper is a narrative, not a systematic review, aiming to offer a current, clinically applicable overview of diagnostic tools for invasive candidiasis and to facilitate clinical decision making.

## 3. Results

### 3.1. Culture Biomarkers: Blood Cultures

Although considered the “gold standard” for diagnosing invasive fungal infections, blood cultures demonstrate suboptimal sensitivity, around 50%, which means that half of the cases may go undetected. This limited sensitivity that ranges between 21 and 71% can be attributed to intermittent or low-level fungemia, as well as deep-seated infections where *Candida* species are rarely detected in the bloodstream [6,7,14].

The volume of blood collected and incubated has a direct impact on pathogen recovery. Current microbiological guidelines recommend inoculating approximately 20 mL of blood per culture vial in adult patients, with a maximum incubation period of five days [6,7,15].

Most commercially available blood culture systems can support fungal growth. Becton Dickinson (BD), one of the most widely used systems, produces the BD BACTEC Mycosis Bottles, which are specially designed for the detection of fungal pathogens. Another automated microbial detection system, the BacT/Alert (bioMérieux, formerly Organon Teknika Corp., Durham, North Carolina, USA) utilizes colorimetric CO_2_ detection produced by growing microorganisms. The BacT/Alert FA Plus bottles are suitable for the recovery and detection of aerobic microorganisms-bacterial or yeasts [16,17].

A comparative study by Tomazin et al. found that BD BACTEC aerobic bottles performed similarly to BD BACTEC Mycosis bottles for the recovery of fungal pathogens, although the dedicated Mycosis bottle showed superior performance for *Aspergillus* and *Cryptococcus* species. In contrast, anaerobic bottles were ineffective for fungal growth in most cases [17,18,19].

Chromogenic media are used in some laboratories as a rapid, budget friendly, preliminary screening tool for differentiating *Candida* species based on species-specific color reactions. These media incorporate biochemical substrate that trigger species-specific colony colors—such as green-blue for *C. albicans*, metallic blue with pink halo for *C. tropicalis*, or pink, rough colonies for *Pichia kudriavzevii*—and may display light blue colonies for *C. auris*, although considerable variability exists among clades. Although chromogenic cultures cannot replace standard culture-based identification or provide antifungal susceptibility data, their accuracy for distinguishing common *Candida* species has been reported as moderate-to-high. Therefore, while not definitive diagnostic tools, chromogenic media complement culture workflows in laboratories [20,21].

Identification obtained from a positive blood culture can be performed using a variety of commercial or automated systems. Matrix Assisted Laser Desorption/Ionization-Time-Of Flight (MALDI-TOF) mass spectrometry enables rapid and accurate identification of fungal species and is considerably faster and less laborious than conventional phenotypic methods. Traditional identification methods may require up to 18 h, compared to MALDI-TOF that provides reliable species identification in approximately 20 min [18,19].

Characterization of *Candida* species from positive blood cultures can be achieved using PCR-based techniques. BIOFIRE ^®^ FILMARRAY ^®^ (bioMérieux, Salt Lake City, Utah, USA) is a Food and Drug Administration (FDA)-cleared multiplex PCR system that integrates sample preparation, amplification, detection, and data analysis in a single workflow. The BIOFIRE BCID2 Panel aims to investigate 43 microorganisms linked to bloodstream infections, covering Gram negative and positive bacteria and yeast (*C. albicans*, *Nakaseomyces glabratus*, *Pichia kudriavzevii*, *C. parapsilosis*, and *C. tropicalis*) along with ten antimicrobial resistance genes in one test [22,23].

The Molecular Mouse platform (MM) (Alifax, Polverara, Padova, Italy) is a qualitative, non-automated, in vitro diagnostic system designed to assist identification of infectious pathogens from a positive blood culture. The platform consists of a compact thermocycler, ready to use cartridges with lyophilized reagents for multiplex real-time PCR, and integrated software for automated data analysis. It analyzes DNA targets using disposable silicon-based devices The MM platform performs deoxyribonucleic acid (DNA) target sequences analysis using disposable lab on chip devices based on Silicon technology. Among the five available tests, MM yeast Blood is dedicated to *Candida* diagnosis, providing results within 1 h after a positive Gram smear. It can identify the following species: *C. albicans*, *Nakaseomyces glabratus*, *Pichia kudriavzevii*, *C. parapsilosis*, *C. tropicalis*, *C. auris*, *C. lusitaniae*, *C. dubliniensis*, *Meyerozyma guilliermondii*, formerly named *C. guilliermondii* [23].

Blood cultures remain the confirmatory, accessible, and easy-to-perform diagnostic method for invasive candidiasis. Unlike bacterial pathogens isolated from blood cultures, which often require careful interpretation to exclude potential contaminants originating from skin flora, particularly in the cases of Gram positive cocci, any fungal growth in blood cultures is clinically significant and require immediate therapeutic action. According to the current guidelines, repeated blood cultures should be obtained on the subsequent days after the initial positive result, and the minimum duration of effective antifungal therapy should be calculated from the first negative blood culture [6].

### 3.2. Non-Culture Biomarkers

#### 3.2.1. Molecular Diagnosis

Whether directed at a single target or multiple targets, molecular techniques are undergoing continuous development. Considered an additional tool in diagnosis, their role is increasingly recognized as a complementary technique along culture-based tests. The ability to detect early candidemia even when blood cultures provide negative results, establishing them as a valuable diagnostic assay in clinical settings. Performed directly from whole blood, serum, cerebrospinal fluid (CSF) or from other sterile sites, molecular assays are considered adjunctive tests in early diagnosis and management.

Although PCR assays demonstrate high analytical sensitivity with a short turnaround time, its clinical applicability remains limited due to a lack of standardized protocols, variable assay performance across laboratories, and the absence of formal guidelines recommendation supporting their use as a first-line diagnostic test. Ongoing standardization efforts and multicenter evaluation are needed to clarify their role in future diagnostic algorithms [24,25].

Regardless of their use in clinical practice being still constrained by methodological variability and lack of standardization, PCR-based methods are increasingly recognized in research settings for their ability to rapidly detect fungal DNA (deoxyribonucleic acid) and support early therapeutic decisions [25,26].

##### Bruker’s Fungiplex^®^ Candida IVD PCR Kit

A multiplex real-time PCR test, designated for rapid detection of the most prevalent pathogens associated with invasive candidiasis, provides a rapid result within 3 to 5 h directly from whole blood without requiring a prior positive blood culture. With a reported sensitivity of 98.4% and specificity 99.8%, the Fungiplex *Candida* assay enables early identification of *Candida* species infections. The kit includes three PCRs for detecting *Candida*: pan-*Candida* species PCR (Csp), one specific PCR for detecting *Nakaseomyces glabratus*, and one specific PCR for detection of *Pichia kudriavzevii* [27].

Higher quantification cycles (Cq) values observed in PCR-based assays among patients with negative blood cultures may explain the reduced detection rate of *Candida* species by conventional culture methods. Prophylactic treatment with antifungals may inhibit or delay growth in blood culture but may not negatively influence the performance of the molecular assays [28].

##### Magicplex™ Sepsis Real-Time Test (Seegene Inc, South Korea)

It is a rapid multiplex performed directly from blood that needs DNA extraction and amplification followed by identification, providing results in approximately six hours. Designed for the detection of microorganisms commonly implicated in sepsis, it can identify up to 90 pathogens at the genus level and 27 pathogens at the species level. *Candida* species detection is limited to five species: *C albicans*, *C tropicalis*, *C. parapsilosis*, *Nakaseomyces glabratus* and *P. kudriavzevii* and does not include *C. auris* [29].

Although PCR enables early fungal detection of nucleic acid, serological assays can indirectly indicate infection by detecting host antibody response or fungal cell wall antigens.

#### 3.2.2. Serological Biomarkers

##### Candida Antigen and Antibody Detection

*Candida* antigen and antibody detection assays gained broader acceptance for diagnosing invasive candidiasis, at first in 2016, in the United States of America (USA), following their inclusion in the Clinical Practice Guideline for the Management of Candidiasis by the IDSA [6].

In Europe, mannan antigen and anti-mannan antibody assays are considered among the best studied tests, for diagnosing invasive candidiasis in high-risk hematological patients, being endorsed by the Third Europe Conference on Infections in Leukemia [30].

Numerous diagnostic platforms and commercial laboratories have developed assays designated for the detection of circulating *Candida* antigen and antibodies. The widespread availability of these tests has influenced clinical practice, and their results are now commonly integrated into diagnostic decision making. The performance of these tests remains robust even in immunocompromised patients, in those receiving chemotherapy or stem cell transplantation. This performance is primarily due to their ability to detect fungal components regardless of the host’s neutrophil response [6].

Mannan is a polysaccharide part of the *Candida* cell wall and serves as the primary target of Antigen detection assays. Circulating *Candida* antigen can be detected early in the bloodstream, often several days before positive blood culture, and exhibits rapid clearance after the initiation of effective antifungal therapy, compared to the more persistent antibody response [31].

Anti-mannan antibodies, predominantly IgG class, persist longer than circulating antigens and may indicate prior exposure or ongoing infection to *Candida*, rather than an acute primary episode. It is worth highlighting that a negative mannan antigen does not exclude infection, especially in patients with localized *Candida* disease [14,32,33].

Diagnostic performance has been demonstrated for cutoff values of >125 pg/mL for antigen and >10 antibody units (AU)/mL for the anti-mannan antibody. Combined testing for mannan antigen and anti-mannan antibody using this diagnostic cutoff improves diagnostic sensitivity and provides more reliable evidence of invasive fungal infection when both assays are performed simultaneously [34].

The diagnostic accuracy of mannan antigen assays revealed a sensitivity of 58% (95% CI, 53–62%) and specificity of 93% (95% CI, 91–94%). Similarly, anti-mannan antibodies assay demonstrated a sensitivity of 59% (95% CI, 54–65%) and a specificity of 83% (95% CI, 79–97%). When combined, mannan and anti-mannan testing exhibits superior diagnostic performance with a pooled sensitivity of 83% (95%CI, 79–87%) and a specificity of 86% (95% CI, 82–90%) [30].

In comparison with the conventional diagnostic process, combined serological assays show positivity in more than 73% of patients at least two days earlier, and in some cases, even sooner than the blood cultures becoming positive [35].

Nevertheless, these tests also have several limitations. Broad-spectrum antibiotics can lead to false positive results, both in those with a high *Candida* colonization index or in patients with injury of the mucosal barrier. Differences among *Candida* species can also affect test performance. *C. parapsilosis* and *C. auris* produce lower detectable levels of circulating antigen, which translate into lower sensitivity and can lead to potential diagnostic delays, particularly in cases when diagnosis is critical [14,32,33].

While mannan antigen detection allows early identification, β-D-glucan could provide better fungal coverage.

##### 1-3-β-D-Glucan (BDG)

It is a polysaccharide part of the fungal cell wall that is shed into the bloodstream during fungal proliferation and lysis, particularly in the context of invasive fungal infection, making it a suitable biomarker. Because it can detect a broad spectrum of fungi, including *Candida*, *Aspergillus*, and *Pneumocystis*, is widely applicable for non-culture diagnostics purposes [36,37].

It was first recognized as a nonculture diagnostic biomarker in 2009 at the 3rd Conference in Leukemia, which stated that repeated positive BDG results can support the evidence of invasive fungal infection in patients with neutropenia and clinically suggestive symptoms [38].

The 2019 EORTC/MSG consensus definitions incorporated serological testing as part of the mycological criteria for diagnosing probable invasive fungal infection. BDG, with a cutoff value above 80 ng/L, is used in combination with clinical features and host risk factors, particularly in patients with neoplasia and hematological malignancies. This inclusion underlines the growing role of BDG as a supportive marker for early diagnosis in high-risk populations [4].

The principal strength of BDG testing relies on its high Negative Predictive Value (NPV), which, in clinical practice, can support the decision of withholding or of discontinuing empiric antifungal therapy. In contrast, the utility of positive BDG result for initiating treatment is more limited, primarily due to relatively low Positive Predictive Value (PPV). For diagnosing invasive candidiasis, most studies report an NPV exceeding 90% and a PPV below 70% [39]. Therefore, BDG is most valuable as a rule-out test rather than a confirmatory one.

Meta-analysis suggests that BDG demonstrates a good diagnostic performance, with an overall sensitivity of 75–83% and a specificity of 63–87%. Despite this, intraspecies variability affects assay performance: *C. albicans*, *Nakaseomyces glabratus* and *C. tropicalis* typically exhibit higher levels of BDG compared to *C. auris* and *C. parapsilosis*. In these situations, the lower glucan content may result in false negative results [40,41].

Elevated levels of BDG may also occur in patients undergoing renal replacement therapy, particular in those using cellulose membranes or filters, albumin administration, and broad-spectrum antibiotics, especially in those containing beta-lactamase inhibitors. In such cases, clinicians should carefully take into consideration the possibility of false positive results [39].

The prognostic implication of BDG levels appears distinct: persistently elevated levels on serial measurements are associated with increased mortality, whereas rapid decline correlates with a favorable clinical outcome in invasive candidiasis [42].

### 3.3. C-Reactive Protein, Procalcitonin

C-reactive protein (CRP) and Procalcitonin (PCT) are widely available biomarkers, routinely included in standard diagnostic laboratory workups. These assays can be performed in any hospital setting without the need for specialized training or complex medical equipment.

CRP is an acute phase reactant that is synthesized by the liver, in response to Interleukin 6 stimulation. Serum levels begin to rise within approximately 12 h after inflammatory activation, peak at around 48 h, and remain elevated even after inflammation resolves, making CRP a highly sensitive but nonspecific marker of inflammation [43].

Procalcitonin, a prohormone of calcitonin, released in response to bacterial infection, serves as more accurate marker than CRP for distinguishing systemic bacterial infection from noninfectious conditions [44]. PCT levels are rising more rapidly compared to CRP, typically within six hours, and are particularly useful in differentiating bacterial infection from viral or noninfectious states [45].

In fungal infection, CRP levels are generally higher than PCT. A CRP cutoff value of 9.75 mg/dL and a PCT cutoff value of 0.34 ng/mL have been reported to provide a diagnostic sensitivity of 71% and specificity of 84% for CRP, and 58% and 91% for PCT, respectively. The Positive Predictive Values of 81% and Negative Predictive Values of 75% for CRP, respectively, 86% and 69%, were reported for Procalcitonin [44].

When interpreted alongside BDG and/or a mannan/anti-mannan antibody, these inflammatory markers may aid in distinguishing bacterial from fungal infection etiology in critically ill patients.

## 4. Discussion

Diagnosing invasive candidiasis remains challenging due to its nonspecific clinical presentation in addition to limitations of conventional laboratory methods, which are time-consuming and often provide low sensitivity [46]. As a result, recognizing the clinical scenario and patient profiles at higher risk is essential for enabling early suspicion and timely diagnostic investigation.

Blood cultures continue to represent the “gold standard” for diagnosis. When positive they confirm the infection and identify the causative species, allowing antifungal susceptibility testing, offering essential information for guided antifungal therapy [6,14].

However, their overall sensitivity is limited to approximately 50%. This can be translated into the fact that, while a positive blood culture result is definitive, a negative result does not exclude the possibility of invasive candidiasis [6,14].

Adequate blood sampling is crucial, as insufficient volume or suboptimal collection techniques can further reduce the sensitivity. Therefore, when the total amount of blood available for inoculation is limited, aerobic bottles should be prioritized due to their higher recovery rate for fungal pathogens.

Clinical suspicion of candidiasis is frequent in the immunocompromised population, due to the high burden of disease among them.

Because of the high burden of disease among immunocompromised populations, clinical suspicion of candidiasis is frequent in this group. As a result, EORTC/MSG criteria were developed to standardize diagnosis improving early diagnostic and access to appropriate therapy [4].

In contrast, diagnostic pathways for patients without known immunodeficiency or in critically ill condition remain poorly standardized. To address this gap, the FUNDICU initiative proposed research-based definitions for invasive fungal infections in the ICU setting. Due to insufficient validation and variable diagnostic test performances, their applicability remains limited to research use only [5].

Molecular techniques have gained increasing importance, enabling earlier detection compared with traditional culture-based methods. Although they face technical variability and lack of standardization, PCR-based techniques demonstrate high sensitivity and specificity. The main characteristic of this type of assay is the fact that they can be performed directly from whole blood, without need for a prior positive culture. These qualities make them promising candidates for integration into future diagnostic algorithms [26,34,47].

Mannan antigen and anti-mannan antibody assays can detect *Candida* cell wall components directly from blood and have demonstrated robust performance even in immunocompromised patients. Combined testing enhances sensitivity and may render in positive result even days before cultures become positive, potentially improving the clinical outcome. However, test variability and species-dependent differences, particularly lower antigen levels in *C. parapsilosis* and *C. auris*, can lead to false negative results [30,34].

Similarly, 1-3-β-D-Glucan, a cell wall polysaccharide released during fungal proliferation, is a valuable adjunctive biomarker. Its high Negative Predictive Value allows clinicians to confidently withhold or discontinue empiric antifungal therapy when negative. On the other hand, positive results should be interpreted with caution, given the assay’s moderate specificity and potential for false positive results in patients undergoing hemodialysis, receiving albumin, or treated with beta-lactam antibiotics [39,41].

Inflammatory markers such as C-reactive protein and Procalcitonin remain accessible assays that can complement fungal biomarkers in diagnostic aid. A diagnostic pattern of elevated CRP (>10 mg/dL) combined with a low Procalcitonin (<0.24 ng/dL) may suggest fungal rather than bacterial etiology [46].

Recent evidence suggests that combining culture-based and non-culture-based biomarkers may represent the most promising strategy for timely and accurate diagnosis. For instance, Giacobbe et al. reported that the combination of BDG higher than 80 pg/mL and a PCT lower than 2 ng/mL presented a sensitivity of 66% and specificity of 98% with a PPV of 96% and NPV of 78%, while the combination of CRP higher than 8.5 mg/dL and BDG higher than 80 pg/mL presented a sensitivity of 93.3% and specificity of 79.1% [48,49]. Such diagnostic approaches may improve accuracy and guide early therapeutic decisions.

Despite ongoing progress, significant gaps persist regarding optimal biomarker combinations and their role in non-immunocompromised hosts. Large multicenter studies are needed to validate an integrated diagnostic model and to establish standardized algorithms applicable across different clinical settings.

The characteristics and strengths of both culture-based and non-culture-based biomarkers are summarized in Table 2.

**Figure 1:**
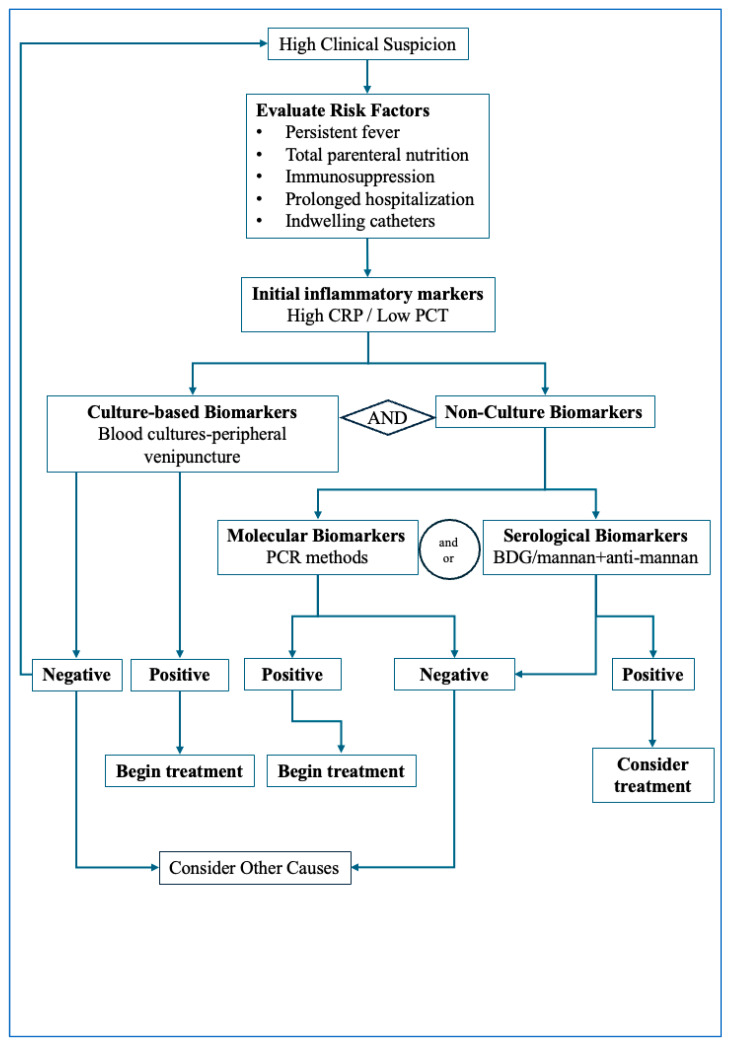
Proposed diagnostic algorithm for investigating invasive candidiasis. Standard inflammatory markers guide early suspicion along with clinical settings, followed by combined culture and non-culture-based biomarkers sampling. While blood cultures take up to 5 days for providing results, rapid non-culture biomarkers assays can guide early therapy decision. This approach emphasizes that treatment initiation should not be delayed pending culture results.

Integrating both culture-based biomarkers and non-culture-based biomarkers may represent the most effective diagnostic strategy for invasive candidiasis. In clinical settings, patients with clinical suspicion of invasive candidiasis that present with high CRP and low PCT levels should undergo prompt blood culture sampling combined with simultaneous non-culture biomarker assessment. This approach may contribute to better diagnostic accuracy, enable earlier therapeutic intervention, and help reduce the overall disease burden. Figure 1 illustrates the proposed diagnostic pathway to be followed in cases of suspected invasive candidiasis. The diagnostic algorithm outlined integrates clinical assessment with sequential biomarker testing to support early detection of invasive candidiasis. The first step involves identifying patients with compatible clinical features—persistent fever despite broad-spectrum antibiotics, unexplained sepsis, or postoperative deterioration—together with major predisposing factors such as ICU stay, indwelling catheters, or immunosuppression. Once suspicion is established, blood cultures should be promptly obtained, while in parallel, rapid non-culture biomarkers (BDG, mannan/anti-mannan or molecular assays) are collected to avoid diagnostic delays. In a low-risk clinical setting, a negative BDG or molecular assay together with persistently negative blood culture may help reasonably exclude invasive candidiasis. In contrast, positive biomarker results should prompt early antifungal therapy. Definitive diagnosis relies on culture or histopathological confirmation, and antifungal treatment should be refined once species identification and susceptibility results become available. This stepwise approach emphasizes early testing and the complementary roles of culture and non-culture biomarkers in guiding timely therapeutic decisions.

This review has several limitations that should be noted. As a narrative review, the main objective is to summarize and interpret clinically relevant aspects from the existing broad literature, and it does not intend to comprehensively identify all published studies; therefore, some data may not have been documented. The evidence base is heterogeneous, with substantial variation in study designs, patient populations, and diagnostic methodologies, which limit direct comparability across studies. These factors should be considered when interpreting the proposed diagnostic pathway.

## 5. Conclusions

Diagnosing invasive candidiasis is challenging except for cases in which blood cultures are positive. More often healthcare providers are relying on indirect or surrogate markers to guide both diagnostic and therapeutic decisions.

Blood cultures remain the reference method for confirming invasive candidiasis, as they provide definitive species identification and antifungal species susceptibility data, but their limited sensitivity and slow turnaround time restrict their value for early diagnosis. Molecular assays, including PCR-based platforms, offer rapid and highly specific detection and may identify infection even when cultures remain negative; however, their implementation is constrained by cost, availability, and limited species coverage.

Serological biomarkers demonstrate variable applicability: mannan and anti-mannan can support early detection, particularly when used in combination, whereas BDG is most useful as a rule-out test due to its high Negative Predictive Value, but must be interpreted carefully given its lower specificity and susceptibility to false positive results. Routine inflammatory markers such as CRP and procalcitonin are not specific for fungal disease but can contribute to differential diagnosis when integrated alongside fungal biomarkers.

Overall, the evidence suggests that no single assay is sufficient for reliable early diagnosis. A combined approach integrating clinical assessment with culture-based and non-culture-based biomarkers offers the greatest diagnostic utility and supports more timely therapeutic decisions. Building on these findings, the principal contribution of this review is the proposed clinician-oriented diagnostic algorithm, which synthesizes current evidence into a practical framework for real-world evaluation of suspected invasive candidiasis. Its stepwise structure aims to support earlier suspicion, guide rational biomarker use, and facilitate more consistent diagnostic pathways across diverse clinical settings.

## Figures and Tables

**Table 1:** Diagnostic criteria including significant risk factors and clinical data associated with invasive candidiasis.

Category	Clinical Details	Source
** Major Risk Factors **	Critical Care and Procedures: Intensive Care Unit stay Central Venous Catheter Total parenteral nutrition Broad-spectrum antibiotics Recent major abdominal surgery Immunosuppression Neutropenia (<500 cells/mm^3^ for >10 days) Hematological malignancy Allogeneic hematopoietic stem cell transplant Solid organ transplant recipient Prolonged corticosteroid use T-cell immunosuppressant therapy Inherited severe immunodeficiency	[4,5,6]
** When to Suspect Candidemia **	▪ Persistent fever unresponsive to broad-spectrum antibiotics ▪ Unknown sepsis or septic shock ▪ Post-abdominal surgery	[5,7]

**Table 2:** Biomarkers comparison.

Biomarker	Turnaround Time	Diagnostic Role	Sensitivity %	Specificity %	Notes	Source
Blood Culture	1–5 days	Confirmatory, Identification, Antifungal susceptibility	50	N/A	Gold standard; requires adequate blood volume; low sensitivity limits utility for ruling out infection	[6,14,15]
β-D-glucan *80 pg/mL cutoff*	~90 min	Early screening	73	80	High Negative Predictive Value, false positive in hemodialysis patients, albumin	[39,41]
Mannan+ Anti-Mannan	~90 min	Adjunct to cultures	83	86	Robust even in immunocompromised patients, lower sensitivity for *C. auris* and *C. parapsilosis*	[30,34]
Fungiplex	3–5 h	Early detection	98.4	99.8	Performed directly from whole blood	[27,28]
CRP + PCT	1–2 h	Supportive only	55–71	84–91	A pattern of high CRP and low PCT suggests fungal rather than bacterial infection	[44]

Note: Reference numbers correspond to the manuscript reference list. Performance metrics are derived from meta-analyses and systematic reviews where available.

## Data Availability

Data are contained in this article, further inquiries can be directed to the corresponding authors.

## Abbreviations

The following abbreviations are used in this manuscript:

ICU	Intensive Care Unit
EORTC/MSGERC	European Organization for Research and Treatment of Cancer and the Mycoses Study Group Education and Research Consortium
FUNDICU	Invasive Fungal Diseases in Adult Patients in Intensive Care Unit
PCR	Polymerase Chain Reaction
ESCMID	European Society of Clinical Microbiology and Infectious Diseases Guidelines
IDSA	Infectious Diseases Society of America Guidelines
ECMM	European Confederation of Medical Mycology
ISHAM	International Society for Human & Animal Mycology
ASM	American Society for Microbiology
BD	Becton Dickinson
MALDI-TOF	Matrix Assisted Laser Desorption/Ionization-Time-Of Flight
FDA	Food and Drug Administration
MM	Molecular Mouse
CSF	Cerebrospinal Fluid
DNA	Deoxyribonucleic acid
Cq	Quantification cycles
USA	United States of America
IgG	Immunoglobulin G
AU	Antibody units
BDG	1-3-β-D-Glucan
NPV	Negative Predictive Value
PPV	Positive Predictive Value
CRP	C-reactive protein
PCT	Procalcitonin

## References

[1] Thambugala K.M., Daranagama D.A., Tennakoon D.S., Jayatunga D.P.W., Hongsanan S., Xie N. (2024). Humans vs. Fungi: An Overview of Fungal Pathogens against Humans. Pathogens.

[2] Yapar N. (2014). Epidemiology and risk factors for invasive candidiasis. Ther. Clin. Risk Manag..

[3] Rüping M.J.G.T., Vehreschild J.J., Cornely O.A. (2008). Patients at high risk of invasive fungal infections: When and how to treat. Drugs.

[4] Donnelly J.P., Chen S.C., Kauffman C.A., Steinbach W.J., Baddley J.W., Verweij P.E., Clancy C.J., Wingard J.R., Lockhart S.R., Groll A.H. (2020). Revision and Update of the Consensus Definitions of Invasive Fungal Disease From the European Organization for Research and Treatment of Cancer and the Mycoses Study Group Education and Research Consortium. Clin. Infect. Dis..

[5] Bassetti M., Giacobbe D.R., Agvald-Ohman C., Akova M., Alastruey-Izquierdo A., Arikan-Akdagli S., Azoulay E., Blot S., Cornely O.A., Cuenca-Estrella M. (2024). Invasive Fungal Diseases in Adult Patients in Intensive Care Unit (FUNDICU): 2024 consensus definitions from ESGCIP, EFISG, ESICM, ECMM, MSGERC, ISAC, and ISHAM. Intensive Care Med..

[6] Pappas P.G., Kauffman C.A., Andes D.R., Clancy C.J., Marr K.A., Ostrosky-Zeichner L., Reboli A.C., Schuster M.G., Vazquez J.A., Walsh T.J. (2015). Clinical Practice Guideline for the Management of Candidiasis: 2016 Update by the Infectious Diseases Society of America. Clin. Infect. Dis..

[7] Cornely O.A., Sprute R., Bassetti M., Chen S.C., Groll A.H., Kurzai O., Lass-Flörl C., Ostrosky-Zeichner L., Rautemaa-Richardson R., Revathi G. (2025). Global guideline for the diagnosis and management of candidiasis: An initiative of the ECMM in cooperation with ISHAM and ASM. Lancet Infect. Dis..

[8] Delaloye J., Calandra T. (2014). Invasive candidiasis as a cause of sepsis in the critically ill patient. Virulence.

[9] Xess I., Pagano L., Dabas Y. (2022). Invasive Fungal Infections 2021. J. Fungi.

[10] Cavalheiro M., Teixeira M.C. (2018). Candida Biofilms: Threats, Challenges, and Promising Strategies. Front. Med..

[11] Vila T., Sultan A.S., Montelongo-Jauregui D., Jabra-Rizk M.A. (2020). Oral Candidiasis: A Disease of Opportunity. J. Fungi.

[12] Kean R., Ramage G. (2019). Combined Antifungal Resistance and Biofilm Tolerance: The Global Threat of *Candida auris*. mSphere.

[13] Fang W., Wu J., Cheng M., Zhu X., Du M., Chen C., Liao W., Zhi K., Pan W. (2023). Diagnosis of invasive fungal infections: Challenges and recent developments. J. Biomed. Sci..

[14] Clancy C.J., Nguyen M.H. (2013). Finding the “Missing 50%” of Invasive Candidiasis: How Nonculture Diagnostics Will Improve Understanding of Disease Spectrum and Transform Patient Care. Clin. Infect. Dis..

[15] Michael L., Wilson M. (2022). M47 Principles and Procedures for Blood Cultures.

[16] Thorpe T.C., Wilson M.L., Turner J.E., DiGuiseppi J.L., Willert M., Mirrett S., Reller L.B. (1990). BacT/Alert: An automated colorimetric microbial detection system. J. Clin. Microbiol..

[17] Tomazin R., Pliberšek T., Oštrbenk Valenčak A., Matos T. (2023). Different BD BACTEC^TM^ Blood Culture Bottle Types for the Detection of Fungi in Simulated Sterile Body Fluid Samples. Diagnostics.

[18] Afifi A.H.M., Ateya R.M.M. (2021). Evaluation of Simple In-house Method for Direct Microbial Identification of Positive Blood Culture by MALDI-TOF Technology. Int. J. Curr. Microbiol. Appl. Sci..

[19] Dai Y., Xu X., Yan X., Li D., Cao W., Tang L., Hu M., Jiang C. (2021). Evaluation of a Rapid and Simplified Protocol for Direct Identification of Microorganisms From Positive Blood Cultures by Using Matrix Assisted Laser Desorption Ionization Time-of-Flight Mass Spectrometry (MALDI-TOF MS). Front. Cell Infect. Microbiol..

[20] Horvath L.L., Hospenthal D.R., Murray C.K., Dooley D.P. (2003). Direct Isolation of Candida spp. from Blood Cultures on the Chromogenic Medium CHROMagar Candida. J. Clin. Microbiol..

[21] (2022). CHROMagar™ Candida Plus. Instructions for Use. NT-EXT-115, Version 4.0. CHROMagar, Paris, France. https://www.chromagar.com.

[22] bioMérieux S.A. BIOFIRE^®^ FILMARRAY^®^ TORCH. https://www.biomerieux.com/corp/en/our-offer/clinical-products/biofire-filmarray-torch.html.

[23] Molecular Mouse (Real-Time PCR System). Product Information. Molecular Biology—Alifax S.r.l. https://www.alifax.com/products/molecular-mouse/.

[24] Camp I., Manhart G., Schabereiter-Gurtner C., Spettel K., Selitsch B., Willinger B. (2020). Clinical evaluation of an in-house panfungal real-time PCR assay for the detection of fungal pathogens. Infection.

[25] Arvanitis M., Anagnostou T., Fuchs B.B., Caliendo A.M., Mylonakis E. (2014). Molecular and nonmolecular diagnostic methods for invasive fungal infections. Clin. Microbiol. Rev..

[26] Lamoth F., Clancy C.J., Tissot F., Squires K., Eggimann P., Flückiger U., Siegemund M., Orasch C., Zimmerli S., Calandra T. (2020). Performance of the T2Candida Panel for the Diagnosis of Intra-abdominal Candidiasis. Open Forum Infect. Dis..

[27] Colosimo C., Montanari M.S., Marzucco A., Gatti G., Puccini L., Grumiro L., Dionisi L., Brandolini M., Ingletto L., Guerra M. (2025). Evaluation of molecular mouse sepsis panel: New portable and rapid tests for microorganism detection in suspected blood stream infection. Front. Cell Infect. Microbiol..

[28] Makristathis A., Harrison N., Ratzinger F., Kussmann M., Selitsch B., Forstner C., Hirschl A.M., Burgmann H. (2018). Substantial diagnostic impact of blood culture independent molecular methods in bloodstream infections: Superior performance of PCR/ESI-MS. Sci. Rep..

[29] Won E.J. (2024). Current nonculture-based diagnosis of candidemia. Ann. Clin. Microbiol..

[30] Mikulska M., Calandra T., Sanguinetti M., Poulain D., Viscoli C. (2010). The use of mannan antigen and anti-mannan antibodies in the diagnosis of invasive candidiasis: Recommendations from the Third European Conference on Infections in Leukemia. Crit. Care.

[31] Ellepola A.N.B., Morrison C.J. (2005). Laboratory diagnosis of invasive candidiasis. J. Microbiol..

[32] Clancy C.J., Nguyen M.H. (2018). Diagnosing Invasive Candidiasis. J. Clin. Microbiol..

[33] Clancy C.J., Nguyen M.-L., Cheng S., Huang H., Fan G., Jaber R.A., Wingard J.R., Cline C., Nguyen M.H. (2008). Immunoglobulin G responses to a panel of Candida albicans antigens as accurate and early markers for the presence of systemic candidiasis. J. Clin. Microbiol..

[34] Held J., Kohlberger I., Rappold E., Grawitz A.B., Häckera G. (2013). Comparison of (1→3)-β-d-Glucan, Mannan/Anti-Mannan Antibodies, and Cand-Tec Candida Antigen as Serum Biomarkers for Candidemia. J. Clin. Microbiol..

[35] Yera H., Sendid B., Francois N., Camus D., Poulain D. (2001). Contribution of serological tests and blood culture to the early diagnosis of systemic candidiasis. Eur. J. Clin. Microbiol. Infect. Dis..

[36] Theel E.S., Doern C.D. (2013). β-D-glucan testing is important for diagnosis of invasive fungal infections. J. Clin. Microbiol..

[37] Karageorgopoulos D.E., Vouloumanou E.K., Ntziora F., Michalopoulos A., Rafailidis P.I., Falagas M.E. (2011). β-D-Glucan Assay for the Diagnosis of Invasive Fungal Infections: A Meta-analysis. Clin. Infect. Dis..

[38] Marchetti O., Lamoth F., Mikulska M., Viscoli C., Verweij P., Bretagne S. (2012). ECIL recommendations for the use of biological markers for the diagnosis of invasive fungal diseases in leukemic patients and hematopoietic SCT recipients. Bone Marrow Transplant..

[39] Lamoth F., Akan H., Andes D., Cruciani M., Marchetti O., Ostrosky-Zeichner L., Racil Z., Clancy C.J. (2021). Assessment of the Role of 1,3-β-d-Glucan Testing for the Diagnosis of Invasive Fungal Infections in Adults. Clin. Infect. Dis..

[40] Hsu A.J., Tamma P.D., Zhang S.X. (2021). Challenges with Utilizing the 1,3-Beta-d-Glucan and Galactomannan Assays To Diagnose Invasive Mold Infections in Immunocompromised Children. J. Clin. Microbiol..

[41] Ullah N., Muccio M., Magnasco L., Sepulcri C., Giacobbe D.R., Vena A., Bassetti M., Mikulska M. (2025). Species-Specific Sensitivity and Levels of Beta-D-Glucan for the Diagnosis of Candidemia—A Systematic Review and Meta-Analysis. J. Fungi.

[42] Carelli S., Posteraro B., Torelli R., De Carolis E., Vallecoccia M.S., Xhemalaj R., Cutuli S.L., Tanzarella E.S., Dell’aNna A.M., Lombardi G. (2024). Prognostic value of serial (1,3)-β-d-glucan measurements in ICU patients with invasive candidiasis. Crit. Care.

[43] Rajab I.M., Hart P.C., Potempa L.A. (2020). How C-Reactive Protein Structural Isoforms With Distinctive Bioactivities Affect Disease Progression. Front. Immunol..

[44] Kokkoris S., Angelopoulos E., Gkoufa A., Christodouli F., Ntaidou T., Theodorou E., Dimopoulou G., Vasileiadis I., Kremmydas P., Routsi C. (2024). The Diagnostic Accuracy of Procalcitonin and Its Combination with Other Biomarkers for Candidemia in Critically Ill Patients. J. Clin. Med..

[45] Liu Y., Zhang X., Yue T., Tang Y., Ke Z., Li Y., Luo X., Huang L. (2022). Combination of C-Reactive Protein and Procalcitonin in Distinguishing Fungal from Bacterial Infections Early in Immunocompromised Children. Antibiotics.

[46] Yeo S.F., Wong B. (2002). Current Status of Nonculture Methods for Diagnosis of Invasive Fungal Infections. Clin. Microbiol. Rev..

[47] Monday L.M., Acosta T.P., Alangaden G. (2021). T2Candida for the Diagnosis and Management of Invasive Candida Infections. J. Fungi.

[48] Giacobbe D.R., Mikulska M., Tumbarello M., Furfaro E., Spadaro M., Losito A.R., Mesini A., De Pascale G., Marchese A., Bruzzone M. (2017). Combined use of serum (1,3)-β-D-glucan and procalcitonin for the early differential diagnosis between candidaemia and bacteraemia in intensive care units. Crit. Care.

[49] Kazancioglu S., Bastug A., Kayaaslan B., Mutlu N.M., Calci E., Turhan T., Mumcuoglu I., Akinci E., Bodur H. (2022). Diagnostic value of β-D-glucan alone or combined with Candida score, colonization index and C-reactive protein for candidemia. J. Infect. Dev. Ctries..

